# Real-World Use of a Decellularized Porcine Placental Extracellular Matrix in Hard-to-Heal Wounds: A Retrospective, Single-Center Study

**DOI:** 10.3390/jcm14196823

**Published:** 2025-09-26

**Authors:** Eddie Soong, Brad Marcinek, Cristin Taylor, Christopher Michaelis

**Affiliations:** 1 Charleston Wound Care, Mount Pleasant, SC 29464, USA; 2 Convatec Limited, CTEC, First Avenue, Deeside Industrial Park, Deeside CH5 2NU, UK

**Keywords:** porcine placental extracellular matrix, hard-to-heal wounds, cellular and/or tissue-based product, real-world data

## Abstract

**Background/Objectives**: Treatment of hard-to-heal wounds remains a challenge in clinical practice. This study aimed to assess the real-world efficacy of a novel porcine placental extracellular matrix (PPECM; InnovaMatrix^®^ AC, Convatec, Memphis, TN, USA). **Methods**: This single-center, retrospective study analyzed consecutive electronic medical records of patients receiving wound care between May 2022 and May 2024. Eligible patients had a hard-to-heal wound that failed to achieve sufficient healing after a minimum of 4 weeks of standard of care treatment, and went on to subsequent management with PPECM. **Results**: Eighty-nine patients (median age, 79 years) were included. The most common etiologies were traumatic wounds (30.3%), venous ulcers (25.8%), surgical wounds (21.3%), and diabetic ulcers (10.1%). Median wound age at first PPECM application was 11.4 weeks (range, 4.0–484.9). The median percent area reduction of the target wound in weeks 4, 12, and 20 was 43.33% (95% CI, 32.92–64.29; n = 75), 100.00% (95% CI, 100.00–100.00; n = 65), and 100.00% (95% CI, 100.00–100.00; n = 58), respectively. Wound closure occurred in 56 (62.9%) patients. The Kaplan–Meier estimate for the median time to complete wound closure was 66 days (95% CI, 55–96; n = 56) and that for the probability of wound closure in weeks 4, 12, and 20 was 0.21 (95% CI, 0.05–0.35), 0.62 (95% CI, 0.40–0.76), and 0.83 (95% CI, 0.31–0.96), respectively. **Conclusions**: Clinically stalled hard-to-heal wounds displayed a clinically relevant reduction in wound area at 4 weeks following treatment with PPECM, with a high probability of wound closure at 12 and 20 weeks after starting treatment. PPECM may support the closure of hard-to-heal wounds that have failed to respond to standard of care treatment.

## 1. Introduction

Hard-to-heal wounds are defined as wounds that do not transition through the normal phases of healing—hemostasis, inflammation, proliferation, and remodeling [1]—in a reasonable time (e.g., within 4 weeks) after appropriate standard-of-care (SoC) treatment [2,3,4]. They encompass a heterogenous group of etiologies, including venous leg ulcers (VLUs), diabetic foot ulcers (DFUs), pressure ulcers, arterial ulcers, skin disorders, surgical infections, traumatic wounds, and others [5].

A meta-analysis of observational studies reported that hard-to-heal wounds had an estimated total global prevalence of 2.21 per 1000 population [6]. In the United States, the prevalence of hard-to-heal wounds among Medicare beneficiaries increased from 8.2 million (14.5%) in 2014 to 10.5 million (16.4%) in 2019, with the largest increases observed in those <65 years of age [5].

Patients with hard-to-heal wounds often have underlying comorbid conditions and complications, including diabetes, obesity, vascular disease, critical infection, and chronic inflammatory disorders [4]. These comorbidities contribute to an adverse pathological environment that delays wound healing via multiple processes including hypoxia, micro- and macroangiopathy, venous stasis, and bioburden [4]. These processes create a wound environment that is hostile to the healing process, characterized by the dominance of proinflammatory factors, including tumor necrosis factor α, and an imbalance of cytokines such as interferon γ and interleukins 1 and 6 [1]. Mast cells are the major regulators of wound healing. Although they support most key stages of the wound healing process in acute wounds, they can also contribute to a persistent inflammatory state in hard-to-heal wounds, where they increase in numbers and degranulation index, and expression of proinflammatory factors [7,8,9]. For example, in patients with diabetes for whom wound healing is often compromised, mast cell degranulation has been linked to impaired healing, and their reduced expression of vascular endothelial growth factor could translate to impaired angiogenesis, epithelialization, and collagen deposition in the wound [9]. These wounds also feature a prolonged presence of neutrophils and M1 macrophages and an associated overexpression of collagenase and matrix metalloproteinases. Although these enzymes have physiological roles in normal tissue remodeling, in excess they damage the native extracellular matrix (ECM), resulting in an attenuation of cellular migration and proliferation, which manifests in the clinic as delayed wound progression and deficient re-epithelialization [1,10,11].

During the normal wound healing process, individual ECM components native to the host support its many stages, including hemostasis and protein and cell assembly (fibronectin); fibroblast and keratinocyte migration, re-epithelialization, and neovascularization (collagen); tissue remodeling, scaffolding, and epidermal regeneration (laminin); basement membrane assembly and remodeling, as well as keratinocyte survival and differentiation (perlecan, or heparin sulfate proteoglycan 2) [10]. This suggests that ECM plays an essential role in all aspects of the normal wound healing process as a supportive environment for infiltrating host cells, scaffold for new tissue, and source of signals conducive to cell proliferation and differentiation.

Therapeutic ECMs are three-dimensional scaffolds comprising various structural macromolecules supportive of the wound healing process. These molecules are understood to play a supportive role in the body’s native polarization of immune response toward a regulatory, pro-remodeling phenotype and progenitor cell recruitment and differentiation [12]. Sicari et al. demonstrated that ECM components promote M2 macrophage polarization and progenitor cell migration, which may translate into the reduction in the proinflammatory signaling in the wound [13]. Huleihel et al. confirmed downregulation of genes associated with a proinflammatory profile in macrophages in response to degraded ECM in preclinical models [14]. Paige et al. reported a decrease in M1/M2 macrophage ratio in response to a porcine urinary bladder ECM in wound samples of patients with diabetes [15].

A porcine placental extracellular matrix (PPECM; InnovaMatrix^®^ AC, Convatec, Memphis, TN, USA) was developed for the management of several types of wounds. PPECM is a terminally sterilized medical device produced from decellularized amnion and chorion tissue and is composed mostly of placenta’s major structural elements, collagen with smaller quantities of fibronectin, laminin, elastin, hyaluronic acid, and sulfated glycosaminoglycans, many of which also play a role in the normal wound healing process. Human-derived placental allografts have long been used in wound healing applications due to their low immunogenicity, antimicrobial and antifibrotic properties, and potential to induce a predominantly anti-inflammatory M2 response in the host tissue [12,16]. Prospective studies show that human placental ECMs are superior to SoC in healing DFUs [17,18,19,20,21,22] and VLUs [23,24], and are a cost-effective adjunct to SoC [25].

Porcine-derived tissues are considered particularly practical due to their cost effectiveness, reproducibility, and availability in larger sizes sufficient for wound coverage, as compared with other ECM sources [26]. The clinical use of porcine-derived ECMs originated in the 1970s [27]. These biomaterials are currently used across a wide variety of clinical applications in addition to wound care, including in artificial heart valves, vascular prostheses, and ligament repair. PPECM is broadly classified as a cellular or acellular matrix-like product, which encompass a wide range of biomaterials, synthetic materials, or biosynthetic matrices that facilitate tissue repair or regeneration through diverse biological pathways [28]. PPECM was designed as an acellular collagen wound dressing to provide a protective barrier that supports the body’s natural healing process. An ex vivo study demonstrated an increased cell infiltration in the PPECM-treated area, which may translate into better tissue remodeling [29].

The present study explores the real-world use of PPECM in the treatment of several types of hard-to-heal wounds. The objective was to determine the change in wound area and wound closure rates of these clinically stalled wounds after advanced wound care treatment with PPECM in a diverse population of patients treated at a single center.

## 2. Materials and Methods

### 2.1. Study Design

This study retrospectively analyzed routinely collected clinical data obtained from electronic medical records (EMR) at a single center in the United States. All patient data were de-identified following the Health Insurance Portability and Accountability Act Safe Harbor method. This study was performed in compliance with the ethical principles of the Declaration of Helsinki. The study protocol (protocol code, WC-23-444; Version 4.0, approved 9 September 2024) was reviewed and approved by the BRANY Institutional Review Board (Lake Success, NY, USA). Reporting adhered to the Strengthening the Reporting of Observational Studies in Epidemiology (STROBE) guidelines for observational data [30].

In the course of the study, patient data were collected from a maximum of 15 clinic visits corresponding to the initial wound visit on record; the visit 4 weeks prior to the initiation of PPECM; all visits that occurred over the duration of PPECM therapy, up to 12 visits; and a final visit at 20 weeks after initiation of PPECM.

Pre-application wound assessments were reviewed to confirm that the patients’ wounds met the definition of “hard-to-heal” described in the inclusion criteria below. These must have occurred at least 4 weeks prior to the patients’ first PPECM application (initial visit). The end of study period occurred when the patient’s target wound reached a resolution irrespective of the outcome or the patient was lost to follow-up.

### 2.2. Participants

Researchers identified all patients treated at the center with PPECM between 1 May 2022 and 31 May 2024. As PPECM is manufactured from porcine placenta, it is contraindicated in patients with sensitivity or allergy to porcine materials; sensitivity or allergy to collagen; or active or latent infection in or around the application site. PPECM is not indicated for use in third-degree burns. Inclusion/exclusion criteria were prespecified in the clinical protocol prior to outcome review (Table 1).

An exception to the above eligibility criteria for defining hard-to-heal wounds was made for 22 patients referred from an external site, who lacked documented pre-treatment wound measurements. Because a ≥50% reduction in wound area could not be confirmed in these patients, eligibility was instead determined via review of the clinical notes in the EMR. Investigators confirmed that these patients’ wounds failed to demonstrate sufficient reduction following ≥ 4 weeks SoC treatment.

### 2.3. PPECM + SoC Application

PPECM was applied in an outpatient setting after sharp debridement with a curette or scalpel, ensuring direct contact with the wound bed and slight overlap beyond the margins. The device was then covered with a non-adherent primary dressing and an appropriate secondary dressing (e.g., Sorbact Superabsorbent [Essity, Stockholm, Sweden], multilayer compression bandage system, or total contact cast) to manage exudate, maintain moisture, and secure the layers in place. Dressings were fixated with adhesive skin closures (Steri-Strips™, 3M Health Care, St. Paul, MN, USA) and left for 7 days unless earlier change was clinically indicated. PPECM is provided as a single-use, sterile wound covering. No antibiotic prophylaxis or reconstructive procedures were performed.

SoC consisted of weekly debridements (when appropriate) and a broad variety of state-of-the art advanced wound care dressings tailored to address the specific needs of the patient and the characteristics of the wound. SoC dressings are aligned with best wound care practices in ensuring sufficient debridement, promoting a moist healing environment, exudate management, protecting the wound, and providing antimicrobial properties when required. Solutions include but are not limited to manuka honey, silver, collagen, enzymatic debridement, and/or negative pressure.

### 2.4. Outcomes

EMR was reviewed to obtain demographics and clinical characteristics at baseline, including age, sex, ethnicity, race, wound age at first application of PPECM, and wound etiology or type.

The primary outcome was the percent area reduction of target wound, calculated as 100 × (Area [baseline]–Area [week 4]/Area [baseline]) %, at 4 weeks. Secondary outcomes were the percent area reduction of target wound at 12 weeks; time to complete wound closure (calculated as the first time point at which the wound is considered closed); and complete wound closure at 4 and 12 weeks (reported as the proportion of patients achieving this outcome at or before the follow-up point). The study’s definition of wound closure required meeting both of the following criteria: a value of 76–100% recorded for percent epithelialization tissue in the Wound Assessment form and the area of the wound (as calculated for the primary outcome) of 0 cm^2^; this definition was adopted because of the categorical nature of percent epithelialization tissue data in the EMR.

Additional, exploratory outcomes were to describe concomitant management (frequency of antibiotic use and of debridement); number of PPECM applications over the treatment course; wound age at first PPECM application; percent area reduction of target wound at 20 weeks; and complete wound closure at 20 weeks (reported as the proportion of patients achieving this outcome at or before the follow-up point).

All serious adverse events (SAEs) were recorded and identified as treatment-emergent if either the start date was on or after the first day of treatment, or if it cannot be established whether the start date was on or after the first treatment. SAEs considered as potentially related to the study device were identified, with these data reviewed with the primary investigator/treating physician for determination of causality and handled via the sponsoring company’s established complaint investigation process.

### 2.5. Statistical Methods

The study was designed to include up to 75 patients who met the inclusion/exclusion criteria and received PPECM treatment within the specified time period. Rather than formally test a prespecified hypothesis, this study provides a descriptive summarization of retrospective data. Consequently, the statistical methods used do not attempt to control for any sources of bias or confounding.

Descriptive statistics were calculated for continuous variables and frequencies and percentages for categorical variables.

For the endpoint of percent area reduction of the target wound, the baseline date for a given wound corresponded to the week 1 visit during first application of PPECM. As the week 4 follow-up visit used for the primary endpoint occurred three weeks post-baseline, it encompassed a predefined visit window ranging from 18 to 24 days post-baseline. If multiple visits occurred within this range, the later visit was selected for analysis (e.g., if patient visits occurred on days 19 and 23, day 23 was used for the statistical analysis). This approach also applied to the 12-week and 20-week assessments of percent area reduction of the target wound, to maintain consistency of data collection and reporting, while simultaneously accommodating real-world scheduling variations.

Time-to-event analyses (i.e., time to wound closure) were conducted using Kaplan–Meier estimates. Subjects that withdrew before wound closure were right-censored at the study-exit date for the purpose of the Kaplan–Meier analysis. The proportion of patients with a time to wound closure ≤4 weeks, ≤12 weeks, and ≤20 weeks is reported along with their 95% confidence interval (CI). The CI was calculated on the log scale using the Hall-Wellner method and back transformed.

Patients were considered withdrawn if their records indicated that they either did not complete the study or the completion status is unknown. Missing data were not imputed.

All statistical calculations were performed using two different programming languages (R Version 3.4.1 or later as one branch and SAS version 9.4 or Python Version 3.11 or later as the second branch) as a diverse self-checking pair. Statistical significance was set at *p* < 0.05.

## 3. Results

All 89 patients screened met the inclusion criteria and were included in the study. The median follow-up time for all subjects was 20 weeks (interquartile range [IQR], 20–20; mean [standard deviation (SD)], 16 [6.5]). A higher number of patients met the inclusion criteria than originally expected. Of these, 67 (75.3%) were included in the analysis. Twenty-two patients (24.7%) were withdrawn from the study due to loss to follow-up (12 [13.5%]), death (1 [1.1%]), or other reasons ((9 [10.1%]); relocated (3 [3.4%]), discharged to other facility (4 [4.5%]), switched products (2 [2.2%])). These patients were followed for a median of 7 weeks (IQR, 3–10; mean [SD], 6 [3]). Patient baseline demographics and wound characteristics are summarized in Table 2.

### 3.1. Primary Outcome

**Figure 1:**
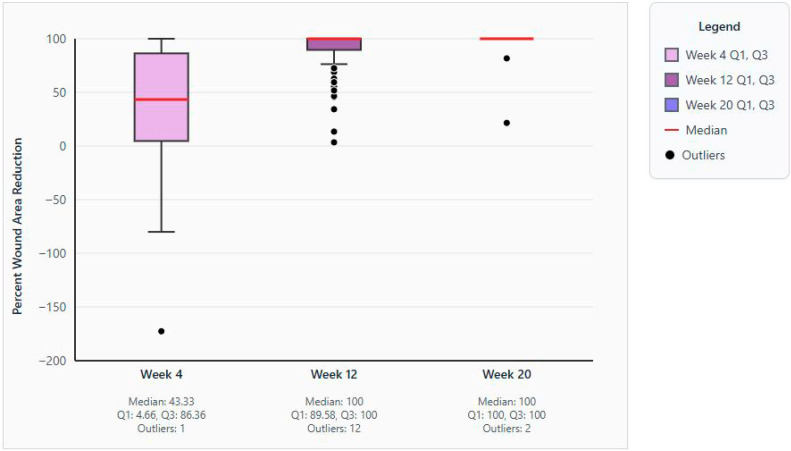
Box and whisker plots of median percent area wound reduction in weeks 4, 12, and 20. Abbreviations: Q1, first quartile; Q3, third quartile.

The median percent area reduction of the target wound in week 4, available for 75 patients, was 43.33% (range, −172.7 to 100.0; 95% CI, 32.92–64.29; Figure 1).

### 3.2. Secondary and Exploratory Outcomes

Wound closure was recorded in 56 (62.9%) patients; in 9 (10.1%) patients the wound did not heal. Two subjects were classified as healed despite failing to meet the 76–100% epithelialization criteria (wound area was 0 cm^2^ at the end of the study for each of these patients). The median percent area reduction of the target wound available for 65 (73.0%) patients in week 12 was 100.00% (range, 3.4–100.0; 95% CI, 100.00–100.00), and that for 58 (65.2%) patients in week 20 was 100.00% (range, 21.5–100.0, 95% CI, 100.00–100.00; Figure 1).

**Figure 2:**
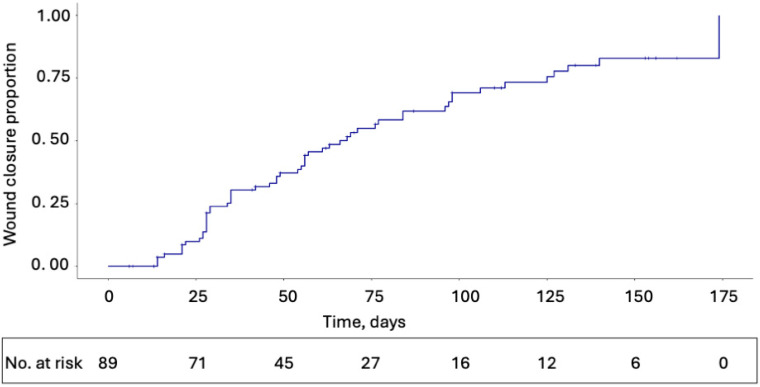
Kaplan–Meier plot for wound closure estimates.

A Kaplan–Meier estimate of the median time to complete wound closure was 66 days (95% CI, 55–96). Kaplan–Meier estimates for probability of wound closure at 4, 12, and 20 weeks were 0.21 (95% CI, 0.05–0.35), 0.62 (95% CI, 0.40–0.76), and 0.83 (95% CI, 0.31–0.96), respectively (Figure 2).

In the course of treatment, patients received a median of 5 (range, 2–13) PPECM applications. Among all 89 patients, a median of 7 (range, 2–15) debridements were performed. Documented medications were available for 65 (73%) of subjects, with no evidence of antibiotic utilization for any subject during the study period.

### 3.3. Safety

There were no SAEs reported among any of the 89 patients.

## 4. Discussion

Wounds treated with PPECM achieved a median 43.33% percent area reduction of the target wound in week 4. These reductions were obtained in a cohort of wounds unresponsive to prior SoC treatment. A recent systematic review found that percent area reduction of ≥40% for VLUs or ≥50% for DFUs in week 4 was predictive of wound healing [31]. This is broadly consistent with our findings, although non-healing traumatic or surgical wounds, which represent two out of three top wound categories in our study (51.6% cumulatively), are not represented wound types in these previous reviews, limiting direct comparison.

In this study wound closure was observed in 62.9% of patients. This is in line with the 57–59% closure rates reported in other real-world studies of viable placental membranes [32,33] and is superior to the mean healing rates of approximately 40% reported at 12 weeks by RCTs assessing the SoC treatment of small, uncomplicated ulcers [34]. Data obtained from the US Wound Registry indicate that the three most common hard-to-heal wound types—DFUs, pressure ulcers, and VLUs—achieve healing rates of just 30.5%, 29.6%, and 44.1%, respectively, at 12 weeks [34]. In our study, probabilities of complete wound closure estimated by the Kaplan–Meier method were 0.21 in week 4 and 0.62 in week 12, which supports favorable clinical performance of PPECM.

Following the use of PPECM, wound closure was achieved in a median of 66 days. Achieving closure of hard-to-heal wounds within a relatively brief timeframe is essential for ensuring optimal outcomes. In a multi-center, international study of 323 hard-to-heal wounds, Hampton et al. reported that the likelihood of complete healing fell from 63.0% for wounds <6 months in duration to 28.4% for those >1 year in duration [35]. Prompt treatment of hard-to-heal wounds with advanced treatment options may reduce morbidity and improve quality of life [36].

Additionally, reducing wound duration may offer an effective means of addressing associated healthcare costs. In the United Kingdom’s National Health Service, the costs to manage wounds that did not heal within a year was approximately 2.5 times greater than those that did heal within this time frame (5.6 billion GBP vs. 2.7 billion GBP, respectively) [37].

The growing prevalence of hard-to-heal wounds is likely to place a tremendous economic burden on an already overtaxed healthcare system. The United States is by far the leading healthcare system globally in terms of estimated wound care spending [38]. In the Medicare beneficiary population alone, conservative estimates are that spending on hard-to-heal wound care costs 22 billion USD annually, which varies depending on wound etiology [5]. However, as treatment of wounds is shared across multiple medical specialties, the actual costs may be underrepresented [39].

These figures highlight the high economic costs associated with chronic wound care. Therefore, cost–benefit analyses, although beyond the scope of the current study, are particularly meaningful and should be conducted for advanced wound care products, such as PPECM, to weigh their projected costs to the healthcare system against their clinical benefit. A recently published study reported that the use of PPECM for the treatment of DFUs was associated with significantly fewer outpatient amputations, episodes of bacteremia, and outpatient hospital visits compared with other cellular, acellular, and matrix-like products in the Medicare fee-for-service population [40].

PPECM offers additional advantages as a porcine-derived, decellularized product. In the United States, PPECM is classified as a medical device. In contrast, dermal matrices and similar products of human origin are classified as human cells, tissues, and cellular and tissue-based products if they meet the US Food and Drug Administration’s criteria for minimal manipulation and homologous use [41]. Comparatively, PPECM’s non-human sourcing results in greater reproducibility, scalable production, and accessibility in the United States as well as in Europe, where restrictions on donation and commercialization of human products limit their use in clinical practice [42].

There were no SAEs observed in the current study. This observation is consistent with recent randomized controlled trials of placental-derived biomaterials in the treatment of hard-to-heal wounds [16]. Based on this, PPECM is expected to have a favorable benefit-to-risk ratio in the management of hard-to-heal wounds.

Real-world analysis is considered a particularly viable approach in wound care research, given the inherent difficulties in enrolling patients in clinical trials who tend to be older and present with multiple comorbidities [43,44]. Relatively higher rates of hard-to-heal wounds are observed in patients with unique considerations (e.g., non-ambulatory, unable to provide self-care) [39] and underlying conditions that traditionally exclude them from participation in clinical trials. This has particularly impacted randomized controlled trials assessing cellular, acellular, and matrix-like products—the broad family of treatments to which PPECM belongs—for the treatment of DFUs and VLUs, which have been limited in their ability to enroll patients presenting with the full spectrum of hard-to-heal wounds [45,46]. As a result, clinicians treating hard-to-heal wounds often must rely on safety and efficacy data derived from populations largely unrepresentative of those they commonly encounter in everyday practice.

In recent years, wound care that was once provided primarily across various medical specialties within hospital settings has shifted towards the approximately 1500 expert wound centers in the United States, where patients receive both SoC and novel treatments [39]. These centers are ideally positioned to address knowledge gaps given their access to large volumes of data on diverse and complicated patient populations. This retrospective analysis draws on data from such a specialty wound care center, thereby meeting a clear unmet need by providing real-world evidence on the use of a novel treatment for hard-to-heal wounds.

### Limitations

The primary limitation of the current study is its retrospective design, which is subject to bias and confounding. The study drew on data collected during routine clinical assessments. This meant that certain relevant demographic and clinical features that can potentially influence wound outcomes (e.g., tobacco use history, body mass index, wound location) were not reported with sufficient consistency allowing for formal analysis. A subset of 22 patients also lacked documented pre-treatment wound measurements. Although EMR documentation identified them as experiencing insufficient wound reduction following a minimum of 4 weeks of SoC treatment, their inclusion potentially introduces further variability into the study population and risk of selection bias; i.e., given the lack of specific wound measurements, it is not possible to confirm that their wounds did indeed fail to improve. Another potential source of selection bias is insurance coverage of ECM-based treatments. Only patients whose health insurance covered those treatments were included in this study and received PPECM. Limited access to advanced wound treatments is a known challenge, which affects both clinical care and research [4]. Additionally, the loss of patients to follow-up through the 20-week period constrained the ability to fully assess the clinical outcomes.

We defined hard-to-heal wounds as those that failed to achieve sufficient healing after a minimum of 4 weeks of SoC. This timeframe was considered objective and clinically meaningful as it aligns with most wound therapy protocols for when advanced therapies should be initiated in such wounds [3,4] and predicts impaired longer-term healing outcomes [2]. However, healing trajectories are dependent on various factors (wound type and size, patient comorbidities) and the definition of hard-to-heal wounds varies in the literature. Finally, data were obtained from a single specialty wound care center, limiting their generalizability to other healthcare sites with differing levels of experience treating hard-to-heal wounds.

## 5. Conclusions

The PPECM product used in this study is the only ECM-based wound dressing in the United States that combines the unique advantages of a medical device—reproducibility and low immunogenicity—with the high ECM component content of the placenta, and it is currently the sole product of its kind cleared by the U.S. Food and Drug Administration. This report offers real-world evidence, crucial in wound research, of the use of this device in a broad range of hard-to-heal wound types.

It reports that hard-to-heal wounds managed with PPECM show a clinically relevant reduction in wound area at 4 weeks, with a high probability of wound closure in weeks 12 and 20. That these findings were obtained across a diverse patient population indicates that PPECM may provide an optimal environment for healing, supporting the closure of the majority of hard-to-heal wounds.

## Figures and Tables

**Table 1:** Pre-defined inclusion and exclusion criteria.

Inclusion criteria
Male and female subjects aged 18 years or older at the time the data were reported.
Patients with a hard-to-heal wound, defined as those that have a reported history of standard of care * that has failed to show improvement (which is defined as ≥50% reduction in wound area over 4 weeks of standard wound care treatment [2]).
Initial wound area that is >0.5 cm^2^ and <25 cm^2^
PPECM applied to target wound for a minimum of two consecutive weekly visits inside a 4-week period.
Patients were compliant with wound protection strategies through treatment period (offloading, compression, etc.).
The target wound is not undergoing active management at the time of data entry.
Exclusion criteria
Wound area showed a ≥50% reported reduction in 4 weeks preceding initial PPECM application.
Cases where PPECM was not applied at a minimum of two consecutive weekly visits inside a 4-week period.
Patients who were non-compliant with additional wound protection strategies (offloading, compression, etc.)
Wound area <0.5 cm^2^ or >25 cm^2^
The target wound is still under active treatment.
Any other reason for exclusion at the discretion of the principal investigator.
Malignant wounds.

Abbreviations: PPECM, placental porcine extracellular matrix. * Standard of care constituted weekly maintenance debridement, when indicated, followed by state-of-the-art advanced wound care tailored to patient needs.

**Table 2:** Patient and wound characteristics (n = 89). Data values are No. (%) unless stated otherwise.

Characteristic	Data Value
Age, years	
Mean (SD)	75.52 (6.15)
Median (IQR)	79.0 (72.50–80.00)
Range	48–≥80
18–29	0
30–39	0
40–49	1 (1.1)
50–59	1 (1.1)
60–69	14 (15.7)
70–79	30 (33.7)
≥80	43 (48.3)
Sex, n	
Male	48 (53.9)
Female	41 (46.1)
Ethnicity, n	
Not Hispanic or Latino	72 (80.9)
Unknown	14 (15.7)
Not reported	3 (3.4)
Race, n	
American Indian or Alaskan Native	2 (2.2)
Asian	1 (1.1)
Black of African American	7 (7.9)
Native Hawaiian or Other Pacific Islander	2 (2.2)
White/Caucasian	64 (71.9)
Unknown	13 (14.6)
Wound age at first application of PPECM, weeks	
Mean (SD)	26.28 (58.65)
Median (Q1–Q3)	11.43 (7.29–20.14)
Range	4.0–484.9
Wound etiology or type, n	
Arterial ulcer	2 (2.2)
Burn	2 (2.2)
Cellulitis	1 (1.1)
Diabetic ulcer	9 (10.1)
Neuropathic	2 (2.2)
Pressure ulcer	1 (1.1)
Radiation wound	3 (3.4)
Surgical wound	19 (21.3)
Trauma wound	27 (30.3)
Venous ulcer	23 (25.8)

Abbreviations: IQR, interquartile range; PPECM, placental porcine extracellular matrix; SD, standard deviation.

## Data Availability

The data presented in this study are available on request from the corresponding author due to restrictions to protect confidential/proprietary information.

## Abbreviations

The following abbreviations are used in this manuscript:

CI	Confidence interval
DFU	Diabetic foot ulcer
ECM	Extracellular matrix
EMR	Electronic medical record
HIPAA	Health Insurance Portability and Accountability Act
PPECM	Porcine placental extracellular matrix
SAE	Serious adverse event
SoC	Standard of care
VLU	Venous leg ulcer
